# Cluster analysis of hotspots and research trends of epirubicin-induced cardiotoxicity: a bibliometric study

**DOI:** 10.3389/fphar.2025.1616162

**Published:** 2025-08-20

**Authors:** Dongning He, Wenjuan Wang, Xigang Luo, Yadi Wang

**Affiliations:** ^1^ Medical Oncology of The Third Affiliated Hospital of Jinzhou Medical University, Jinzhou, Liaoning, China; ^2^ Rehabilitation Department of The First Affiliated Hospital of Jinzhou Medical University, Jinzhou, Liaoning, China; ^3^ Medical Laboratory Department of The Third Affiliated Hospital of Jinzhou Medical University, Jinzhou, Liaoning, China; ^4^ Liaoning Provincial Key Laboratory of Follicle Development and Reproductive Health, Jinzhou, Liaoning, China

**Keywords:** epirubicin, cardiotoxicity, cluster analysis, bibliomrtric, BIB

## Abstract

**Background:**

Epirubicin, a widely used anthracycline, effectively treats various cancers but poses a high risk of cardiotoxicity, leading to heart failure and myocardial dysfunction. This study conducts a cluster analysis to map global research trends in epirubicin-induced cardiotoxicity.

**Methods:**

A literature search was conducted using the Web of Science Core Collection database. Bibliometric tools, including VOSviewer, CiteSpace, and R package “bibliometrix”, were employed.

**Results:**

A total of 673 studies were included in the analysis. Italy, China, and the United States led in publication volume. Unicancer was the most prolific institution. Key research was published in high-impact journals such as *Journal of Clinical Oncology, Annals of Oncology,* and *European Journal of Cancer.* P.F. Conte, J.W. Hopewell, and B. Salvadori were the most influential authors. Cluster analysis identified four research hotspots: mechanisms of cardiotoxicity, clinical applications of chemotherapy regimens, combination therapies and pharmacokinetics, formulation advancements and cardioprotective strategies. In addition, there is a clear cut-off among the strongest citation bursts, with the period from 2004–2013 primarily concentrated on disease treatment. From 2014 onwards, the last 10 years have focused on cardiotoxicity and the underlying mechanisms of cardiotoxicity.

**Conclusion:**

This bibliometric study, based on cluster analysis, identified four research hotspots including mechanisms of cardiotoxicity, clinical applications of chemotherapy regimens, combination therapies and pharmacokinetics, formulation advancements and cardioprotective strategies. Future research directions should prioritize the development of AI-driven risk prediction models, integration of multi-omics biomarkers into clinical workflows, and establishment of international cardio-oncology consortiums to enhance personalized cardioprotective strategies and optimize patient outcomes.

## Introduction

Epirubicin, an anthracycline chemotherapeutic agent, is widely used to treat breast cancer, lymphoma, and various solid tumors (Luiz et al., 2023). Despite its efficacy, epirubicin is associated with a significant risk of cardiotoxicity, which can lead to congestive heart failure, cardiomyopathy, and irreversible myocardial damage (Cardinale et al., 2020). The mechanisms underlying epirubicin-induced cardiotoxicity are complex and involve oxidative stress, mitochondrial dysfunction, and inflammatory pathways (Wallace et al., 2020). Given the widespread clinical use of epirubicin, understanding its cardiotoxic effects is critical for improving patient management, risk stratification, and cardioprotective strategies.

Over the past four decades, research on epirubicin-induced cardiotoxicity has expanded, covering a range of topics including biomarkers for early detection, imaging modalities for cardiac monitoring, and potential cardioprotective interventions. Studies have explored dose-dependent toxicity, genetic predisposition, and molecular pathways that contribute to cardiac dysfunction (Johnson and Keyes, 2025; Vulsteke et al., 2015; Xie et al., 2024). Additionally, the development of liposomal formulations and cardioprotective agents such as dexrazoxane has aimed to mitigate the cardiotoxic effects of epirubicin without compromising its anticancer efficacy (Buchalska et al., 2025; Attia et al., 2016). Despite these advancements, challenges remain in accurately predicting which patients are most susceptible to cardiotoxicity and optimizing risk reduction strategies.

As research on epirubicin cardiotoxicity continues to evolve, identifying key contributors, influential studies, and emerging research trends is essential. Bibliometric analysis, a quantitative method for assessing scientific literature, provides a systematic approach to mapping research trends, evaluating collaboration networks, and highlighting major advancements (Hassan et al., 2021). Although previous bibliometric studies have broadly explored anthracycline-induced cardiotoxicity (Kong et al., 2024), as well as doxorubicin-induced cardiotoxicity (Lin et al., 2023) and its underlying molecular mechanisms (Yarmohammadi et al., 2024), a comprehensive analysis specifically addressing epirubicin remains lacking. This study aims to address this gap by conducting a cluster analysis of global research on epirubicin-induced cardiotoxicity.

## Materials and methods

### Literature search and selection

A literature search was conducted in the Web of Science Core Collection (WoSCC), a leading multidisciplinary database (Ho, 2021). The search terms included: TS = (Epirubicin) AND TS = (Cardiotoxicity OR “Cardiovascular toxicities” OR “Cardiac toxicity” OR “Myocardial toxicity” OR “Heart toxicity” OR “Myocardial injur*” OR “Heart injur*”) (Xiao et al., 2023). The search was limited to publications from 1984 to 2024. Only English-language articles were included, while other document types were excluded. Including reviews, meeting abstracts, proceeding papers, editorial materials, corrections, letters, book chapters, and retracted publications. To ensure consistency, the search was performed on December 31, 2024. Duplicate records were identified and removed using the Web of Science built-in duplicate detection function, supplemented by manual verification based on title, author, and publication year matching. Author disambiguation was performed by replacing duplicate author names in the bibliometrix txt files and unifying repeated author names in the VOSviewer “thesaurus_authors.txt” file to ensure accurate authorship attribution. Extracted data included publication and citation counts, titles, authors, institutions, countries, keywords, and journals for bibliometric analysis.

### Statistical analysis and visualization

Bibliometric analysis was conducted using VOSviewer (1.6.20), CiteSpace (6.3. R1), and the R package “bibliometrix” (4.3.3). VOSviewer and CiteSpace were chosen for their visualization capabilities in network analysis, while bibliometrix enabled comprehensive statistical analysis. This tripartite approach ensures robust data triangulation and multi-dimensional interpretation. All clustering analyses were performed using VOSviewer with the association strength method for optimal visualization and interpretation. To ensure visual clarity and readability, node counts were limited to maximum 200 per visualization, with specific parameters set as follows: country collaboration networks included 39 countries with minimum 3 publications each; institutional collaboration networks comprised 89 institutions with minimum 4 publications each; author collaboration networks contained 190 authors with minimum 3 publications each; journal co-occurrence networks included 55 journals with minimum 3 occurrences each; journal coupling networks comprised 55 journals with minimum 3 coupling connections each; and keyword co-occurrence networks featured 100 keywords with minimum 9 occurrences each. VOSviewer mapped institutional and author collaborations, co-authorship, and keyword co-occurrence networks (van Eck and Waltman, 2010). Node size represented publication or citation volume, colors indicated thematic clusters, and connecting lines depicted collaboration strength. CiteSpace utilized the log-likelihood ratio (LLR) clustering algorithm with time slicing (1 year per slice) and pathfinder network scaling for temporal analysis and keyword burst detection. CiteSpace identified emerging research trends and keyword bursts, using time slicing and pruning techniques to generate keyword co-occurrence timelines. The bibliometrix package employed hierarchical clustering using Ward’s method with Euclidean distance for thematic mapping and conceptual structure analysis. The R package “bibliometrix” analyzed global research trends, extracting metrics including H-index, M-index, and G-index to evaluate researcher impact (Bertoli-Barsotti and Lando, 2017; Hirsch, 2005). Journal influence was assessed using the Journal Impact Factor (IF) and 2023 Journal Citation Reports (JCR) quartiles, with Q1 journals representing the top 25% in their respective fields.

## Results

### An overview of publications

**FIGURE 1:**
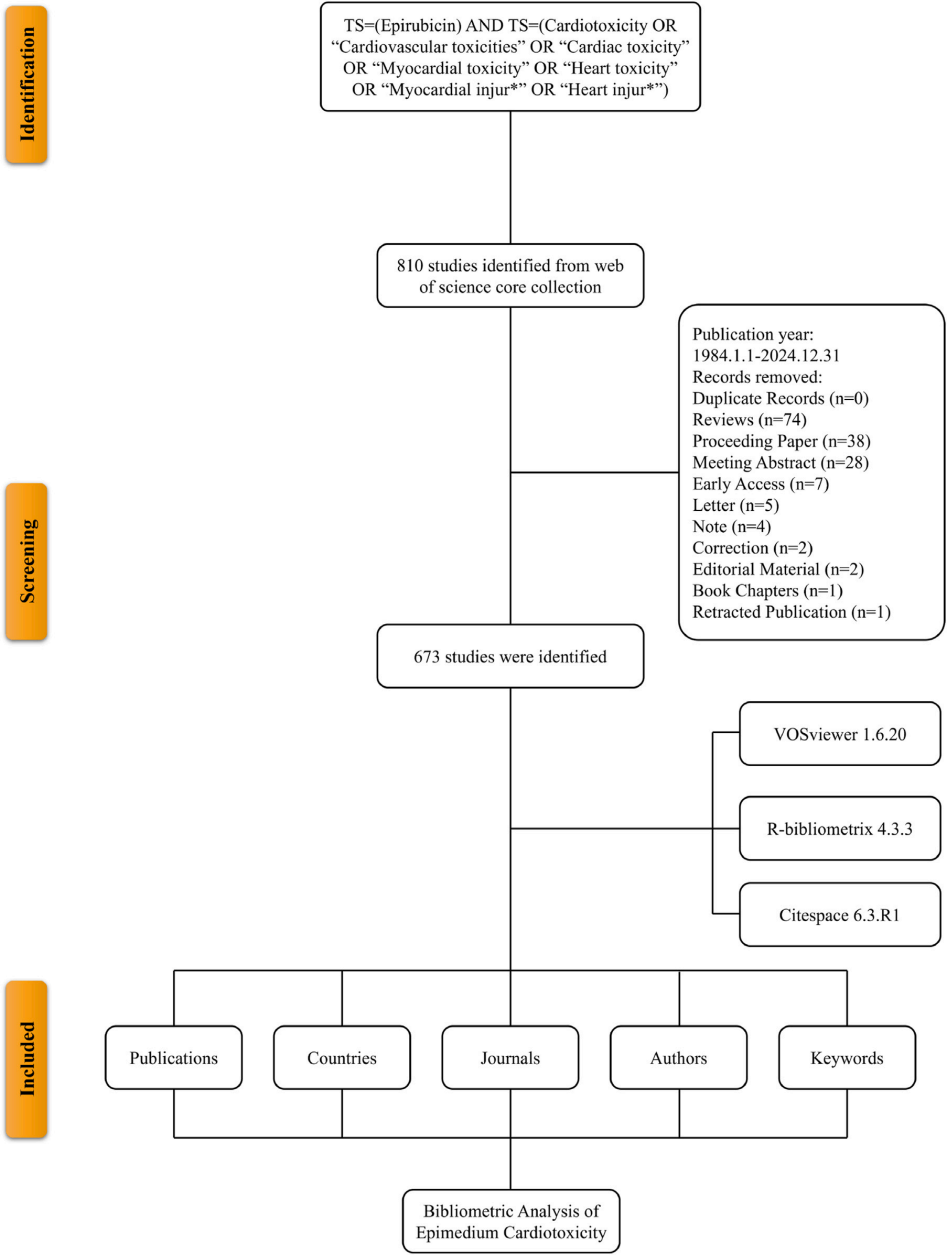
Flowchart of the literature screening process.

**FIGURE 2:**
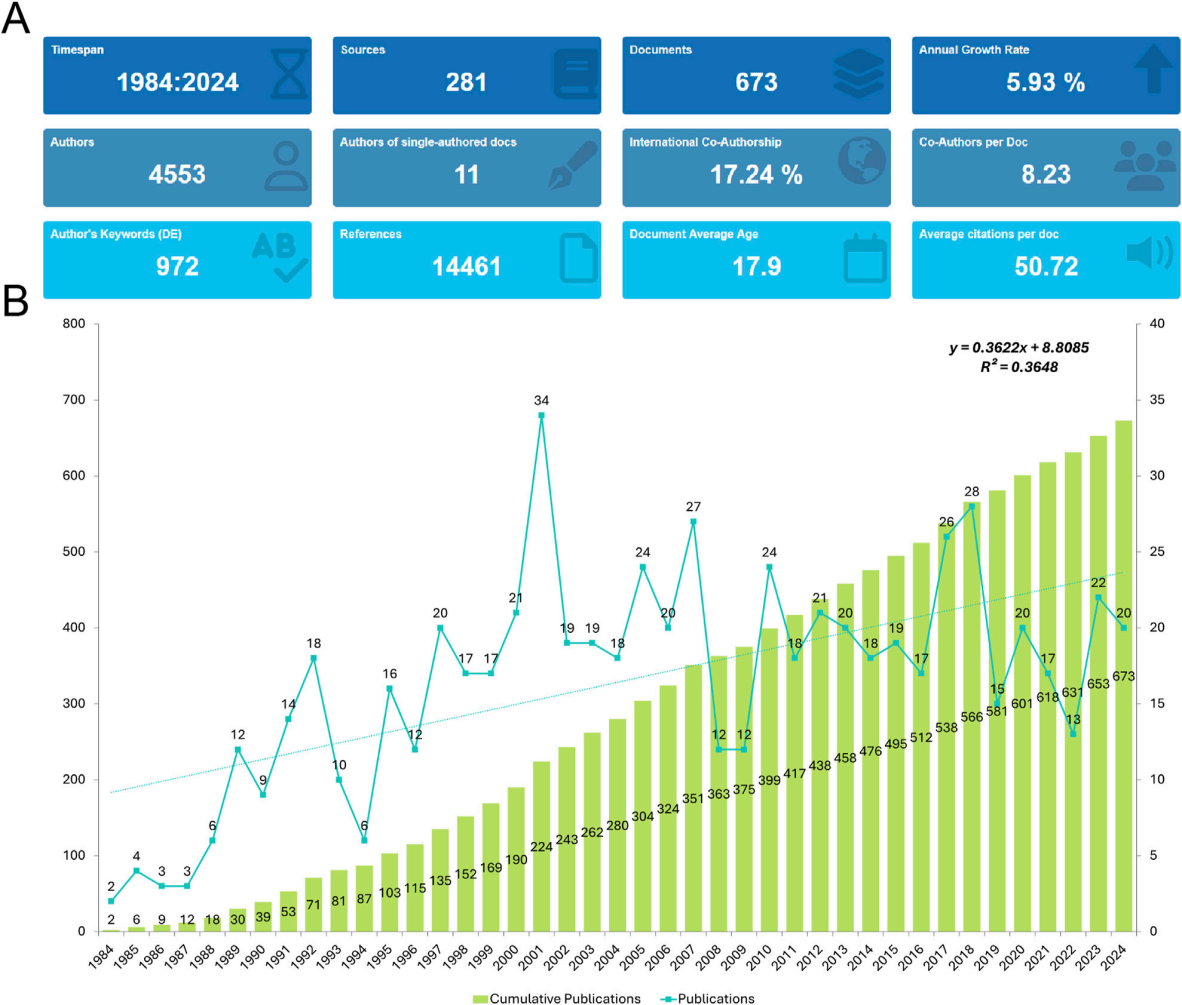
General information. **(A)** Summary Information of the included studies. **(B)** Annual number of publications.

An initial search of the WoSCC retrieved 810 records. After excluding reviews (n = 74), meeting abstracts (n = 28), proceeding papers (n = 38), editorial materials (n = 2), corrections (n = 2), letters (n = 5), book chapters (n = 1), and retracted publications (n = 1), a total of 673 studies published between 1984 and 2024 were selected for analysis (Figure 1). These 673 publications were contributed by 4,553 authors from 2,750 institutions across 65 countries/regions. They were published in 281 journals, citing a total of 14,461 references (Figure 2A). Over the study period, the number of publications exhibited a steady increase, with an annual growth rate of 5.93%. The cumulative publication count reached 673, while annual output fluctuated, peaking at 34 publications in 2001 (Figure 2B).

### Analysis of countries

**FIGURE 3:**
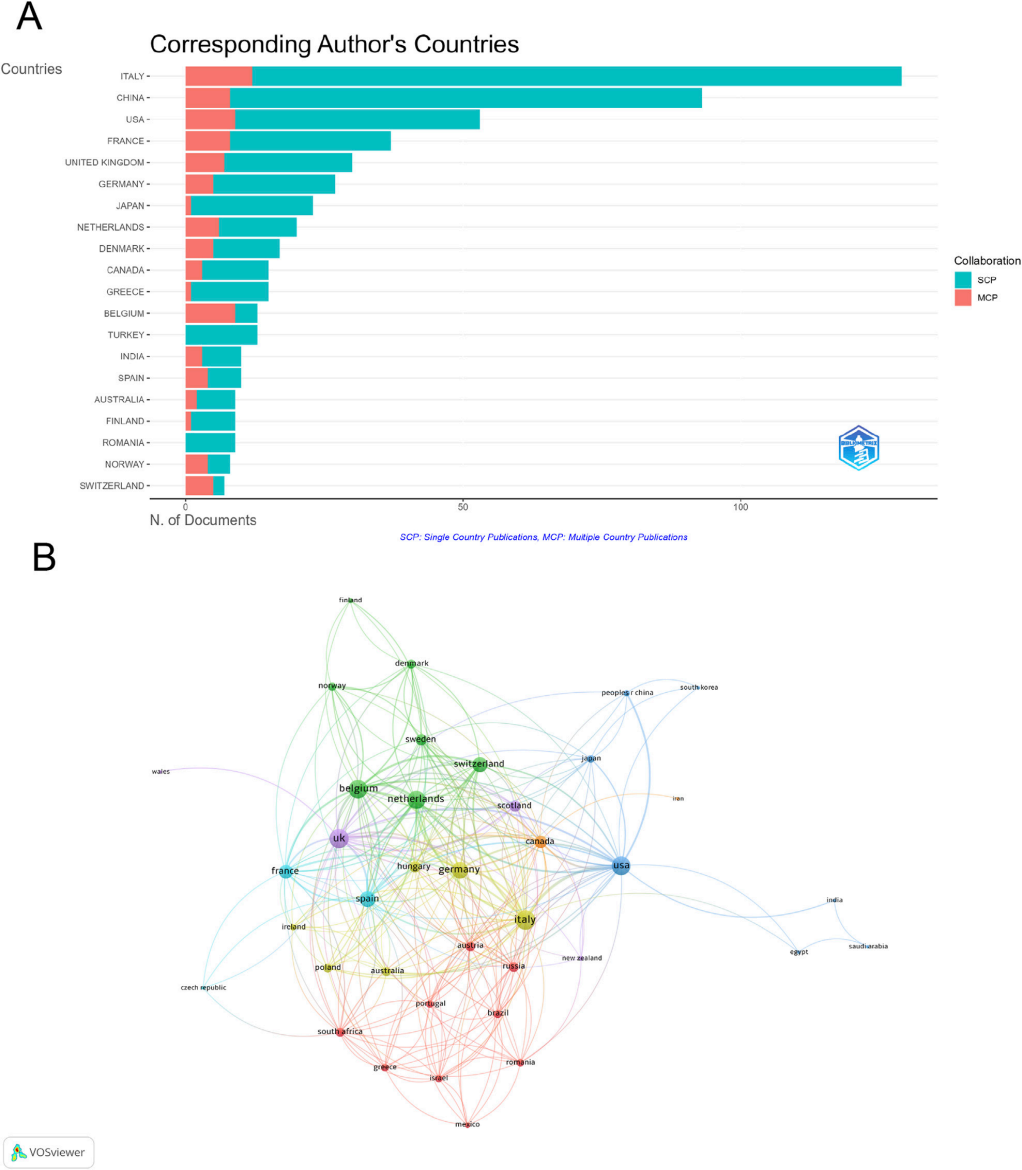
Analysis of countries. **(A)** Distribution of corresponding author’s publications by country. **(B)** Visualization map depicting the collaboration among different countries.

A total of 65 countries/regions contributed to research on epirubicin-induced cardiotoxicity, with the top 20 countries accounting for the majority of publications (Supplementary Table S1; Figure 3A). Italy led in publication volume (129 articles), followed by China (93 articles) and the United States (53 articles). Regarding total citations (TC), the United States ranked first (12,689), while Italy (3,926) and the United Kingdom (3,047) followed. The United Kingdom recorded the highest average citations per article (101.6), with Switzerland (81.6) and Denmark (78.8) also showing significant impact. Among the 39 countries engaged in international collaborations with at least three published articles, Italy (total link strength = 95) had the highest number of partnerships, with the United Kingdom (total link strength = 93) and Belgium (total link strength = 88) ranking second and third respectively (Figure 3B).

### Analysis of institutions

**FIGURE 4:**
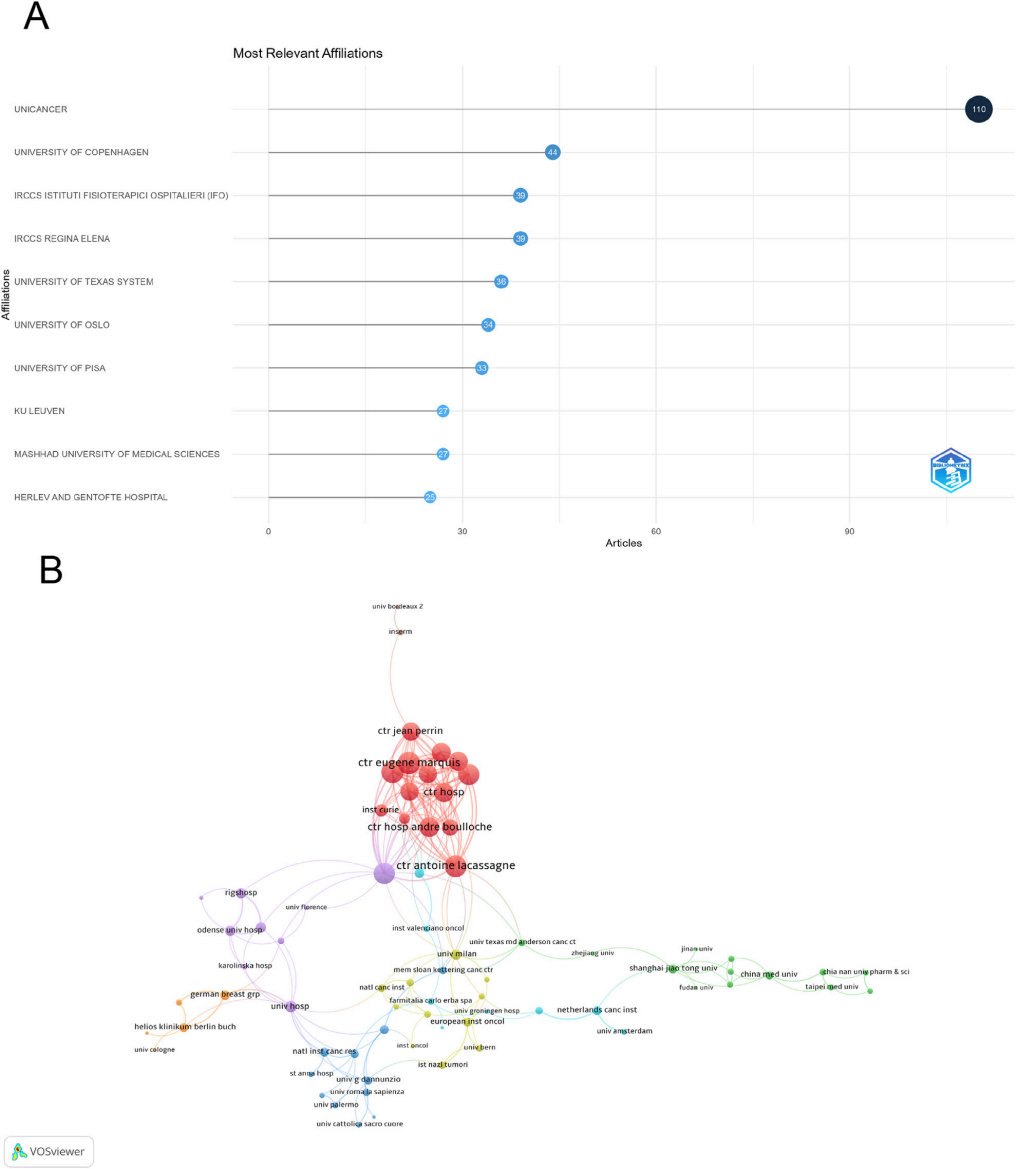
Analysis of institutions. **(A)** Top ten institutions by article count and rank. **(B)** Visualization map depicting collaboration among different institutions.

A total of 2,750 institutions contributed to research in this field, with UNICANCER leading the output with 110 publications, followed by University of Copenhagen (44) and IRCCS Istituto Fisioterapico Ospedaliero (IFO) (Al Khafaji et al., 2025) (Figure 4A). Among 89 institutions with at least four publications, Centre Eugène Marquis and Universitaire du Cancer de Toulouse had the highest number of international collaborations (total link strength = 49), while Centre Antoine Lacassagne (total link strength = 46) ranked second (Figure 4B).

### Analysis of journals

A total of 281 academic journals contributed to publications in this field. The top three journals based on h-index were *Journal of Clinical Oncology* (H-index = 34, IF 2023 = 42.1, TC = 2,786), *Annals of Oncology* (H-index = 24, IF 2023 = 56.7, TC = 699), and *European Journal of Cancer* (H-index = 17, IF 2023 = 7.6, TC = 315). In terms of total publications (TP), the leading journals were *Journal of Clinical Oncology* (TP = 39), *Annals of Oncology* (TP = 35), and *European Journal of Cancer* (TP = 24) (Supplementary Table S2).

**FIGURE 5:**
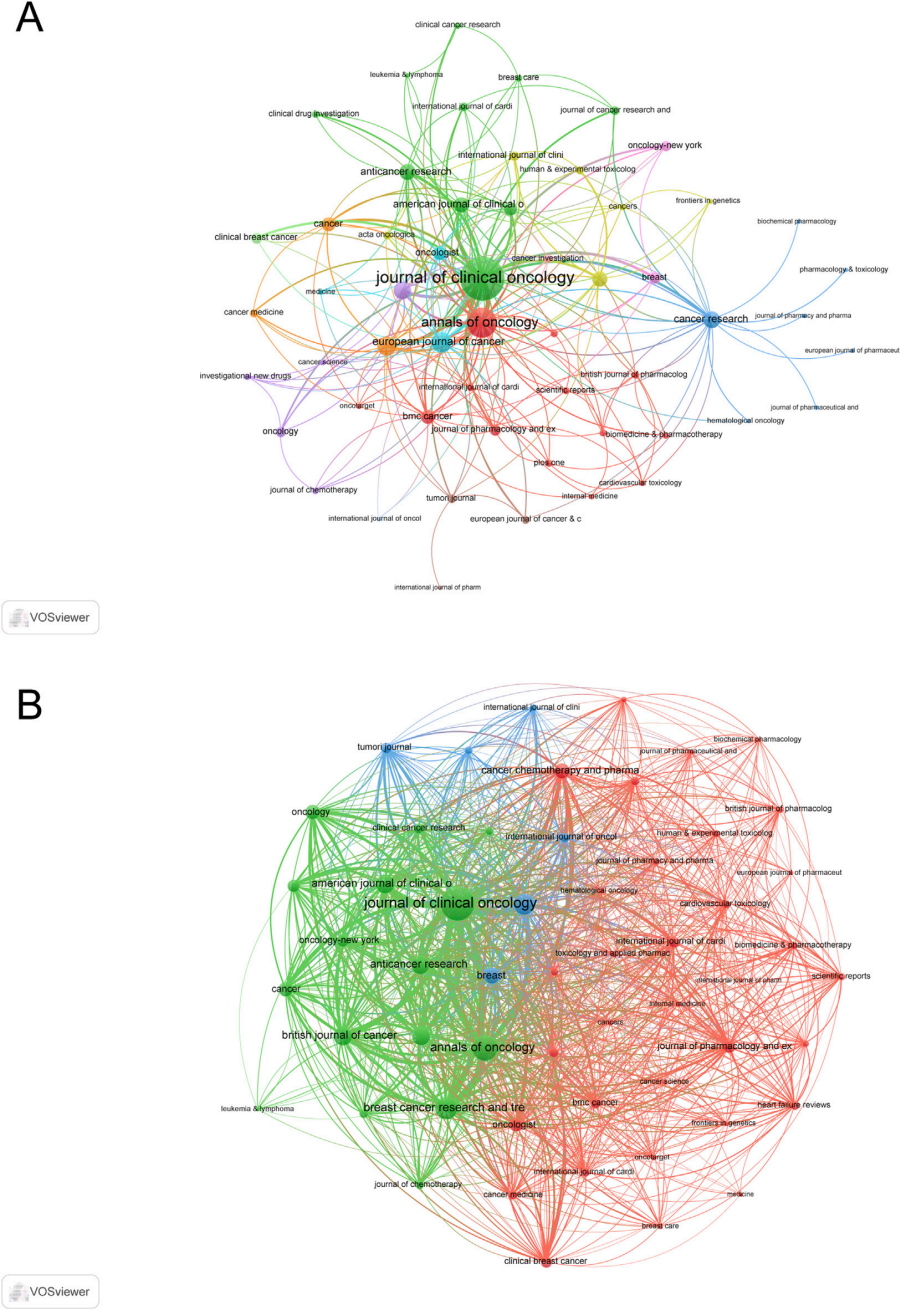
Analysis of journals. **(A)** Co-occurrence Network of Journals. **(B)** Coupling Network of Journals.

The co-occurrence network analysis of journals, which included 55 journals with at least three occurrences, identified *Journal of Clinical Oncology* (total link strength = 370), *Annals of Oncology* (total link strength = 172), and *European Journal of Cancer* (total link strength = 77) (Figure 5A). Similarly, the coupling network analysis, based on 55 journals with at least three coupling connections, highlighted *Journal of Clinical Oncology* (total link strength = 7,145), *Annals of Oncology* (total link strength = 4,048), and *Breast Cancer Research and Treatment* (total link strength = 3,127) (Figure 5B).

### Analysis of authors

**FIGURE 6:**
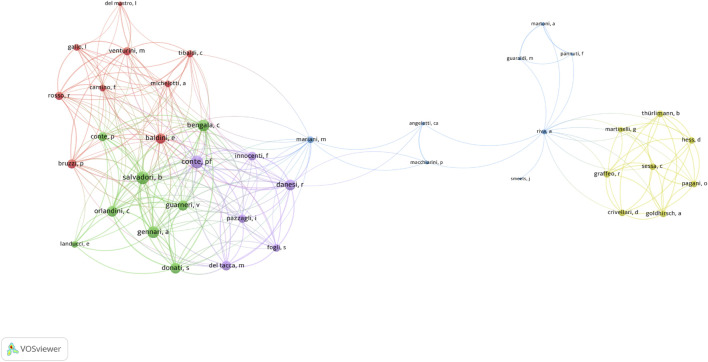
Visualization map depicting the collaboration among different authors.

The author with the highest impact in this field, as indicated by the H-index, was P.F. Conte (H-index = 9, TC = 518). He was followed by J.W. Hopewell (H-index = 8, TC = 247) and B. Salvadori (H-index = 8, TC = 359). Regarding total publications (TP), P.F. Conte also led with 12 articles, followed by A. Gennari (TP = 10, TC = 511) and B. Salvadori (TP = 10, TC = 359). The most cited author was R. Rosso (TC = 1,771) (Supplementary Table S3). Among the 190 authors engaged in international collaborations with at least three articles, P.F. Conte had the highest number of collaborations (total link strength = 78), followed by B. Salvadori (total link strength = 74) and C. Bengala (total link strength = 64) (Figure 6).

### Most cited articles

The analysis of the most cited articles reveals the foundational studies that have shaped research in epirubicin-induced cardiotoxicity. The highest-cited article was published by Slamon et al. in The New England Journal of Medicine (2001), receiving 8,817 citations. This landmark study demonstrated that adding trastuzumab to chemotherapy significantly improved survival and response rates in metastatic HER2-positive breast cancer, while simultaneously highlighting increased cardiotoxicity risks, particularly when combined with anthracyclines. The second most cited article was by O'Brien et al. in Annals of Oncology (2004) with 1,298 citations, which showed that pegylated liposomal doxorubicin offered comparable efficacy to conventional doxorubicin in first-line metastatic breast cancer treatment while significantly reducing cardiotoxicity. The third most cited study was published by Buzdar et al. in Journal of Clinical Oncology (2005) with 941 citations, demonstrating that adding trastuzumab to neoadjuvant chemotherapy significantly increased pathologic complete response rates in HER2-positive operable breast cancer without causing clinical congestive heart failure, though cardiac ejection fraction reductions were observed. These highly cited articles underscore the critical balance between therapeutic efficacy and cardiotoxic risk management in anthracycline-based cancer treatment, establishing the foundation for subsequent research in cardioprotective strategies and alternative formulations.

### Keywords Cluster Analysis

**FIGURE 7:**
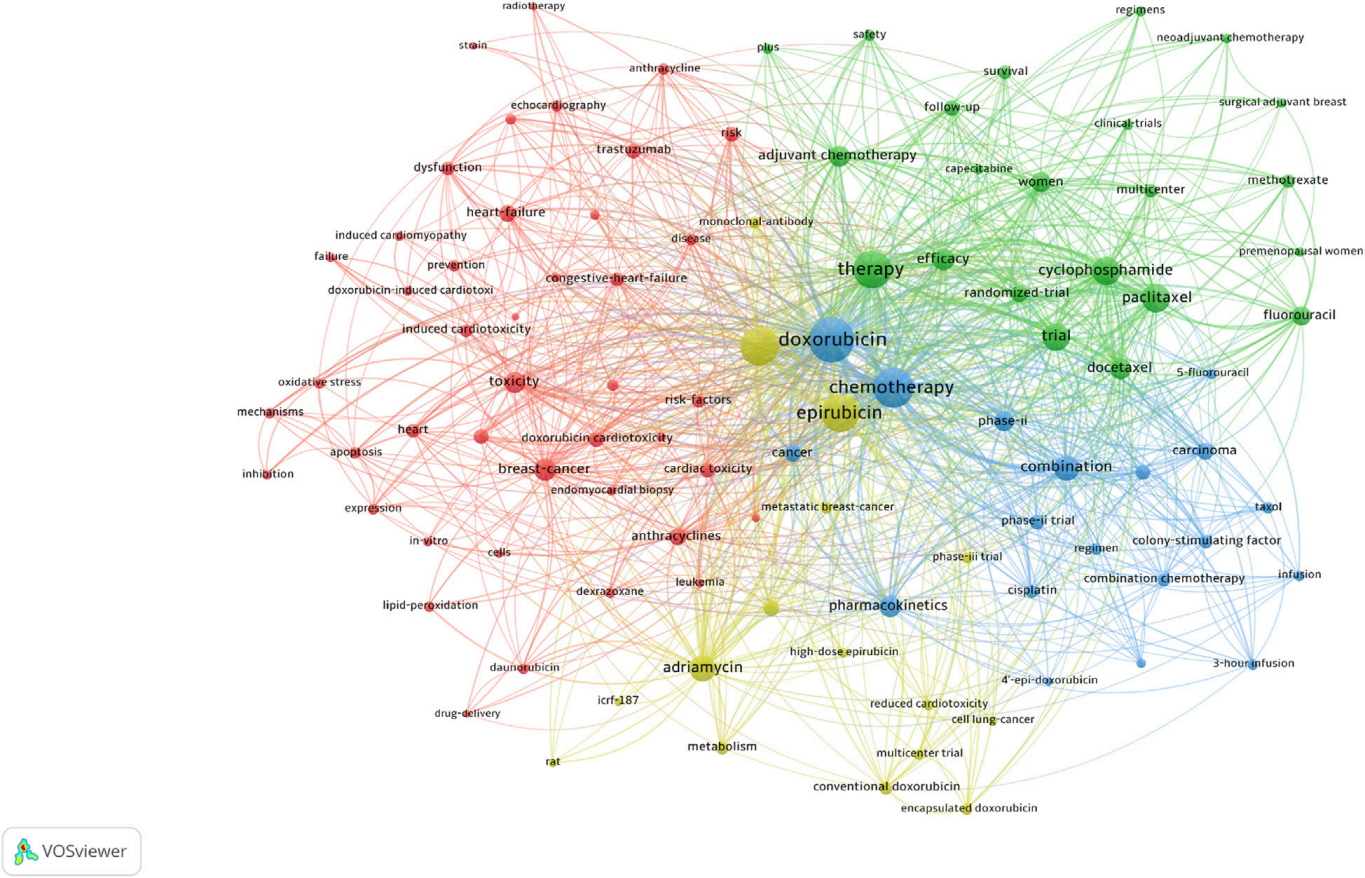
Visual analysis of keyword co-occurrence network analysis.

A total of 100 high-frequency keywords were identified and categorized into four thematic clusters, each reflecting a critical aspect of epirubicin-induced cardiotoxicity (Figure 7). The red cluster centers on the mechanisms of cardiotoxicity, highlighting oxidative stress, apoptosis, and cardiac dysfunction associated with anthracyclines. Key terms include “cardiac toxicity”, “congestive heart failure”, “oxidative stress”, “doxorubicin-induced cardiotoxicity” and “trastuzumab”. The green cluster focuses on the clinical application of chemotherapy regimens, particularly the role of adjuvant and neoadjuvant chemotherapy in cancer treatment. Keywords in this category include “cyclophosphamide”, “paclitaxel”, “randomized trial”, “efficacy”, and “survival”. The blue cluster emphasizes combination therapy approaches, featuring terms such as “combination chemotherapy”, “colony-stimulating factor”, “cisplatin” and “phase II trial”. The yellow cluster pertains to formulation advancements and alternative anthracyclines designed to mitigate cardiotoxicity, with key terms including “adriamycin”, “high-dose epirubicin”, “encapsulated doxorubicin” and “reduced cardiotoxicity”.

### Study trends transition

**FIGURE 8:**
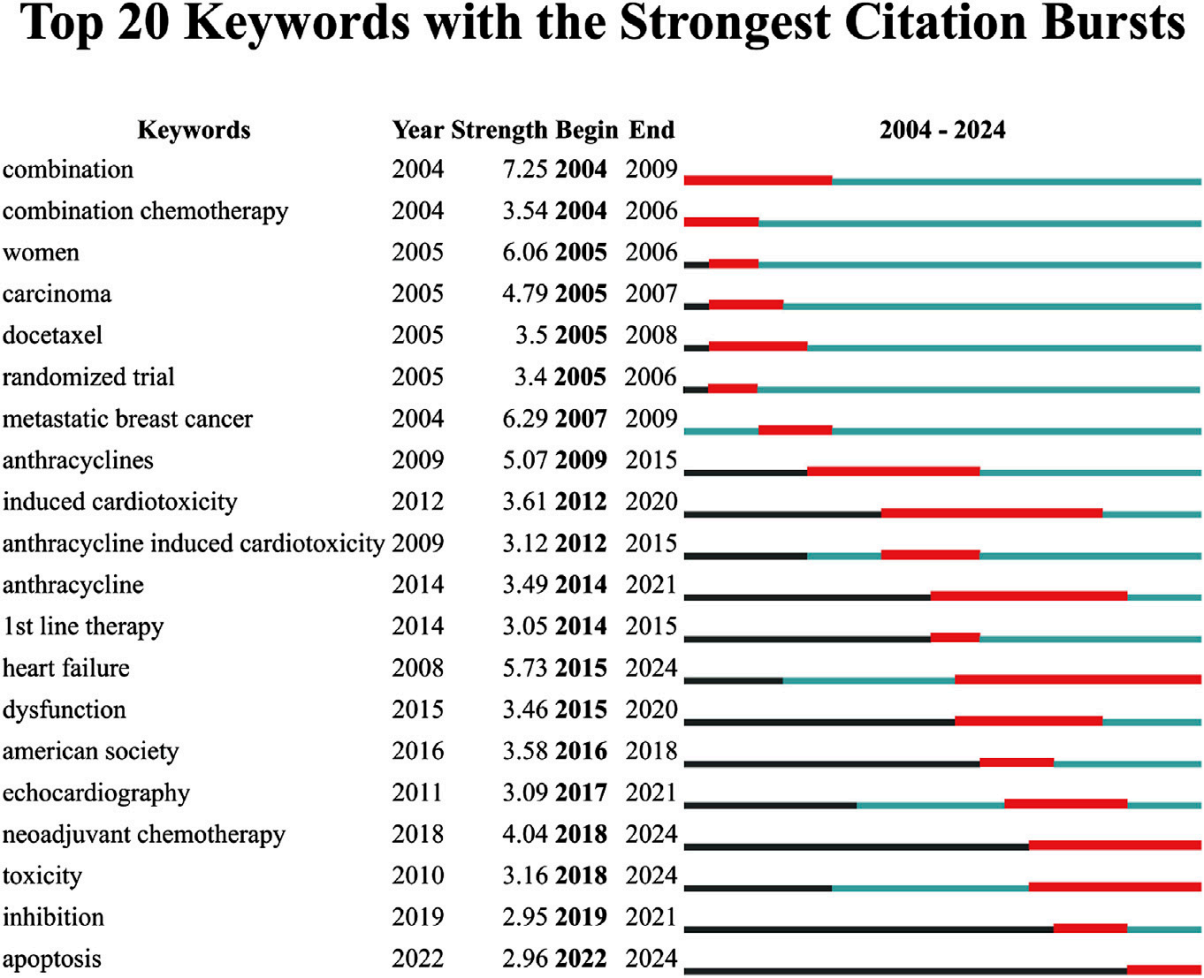
Top 20 keywords with the strongest citation bursts.

The analysis of the top 20 keywords with the strongest citation bursts from 2004 to 2024 reveals shifting research priorities in epirubicin-induced cardiotoxicity (Figure 8). The keyword with the highest burst strength was “combination” (7.25, 2004–2009), and “metastatic breast cancer” (6.29, 2004–2009). Early bursts primarily focused on chemotherapy regimens and oncological treatment strategies, as reflected by keywords such as “combination chemotherapy” (3.54, 2004–2006), “docetaxel” (3.5, 2005–2008), and “randomized trial” (3.4, 2005–2006). In more recent years, the research focus has shifted toward cardiotoxicity mechanisms and mitigation strategies, as demonstrated by emerging keywords such as “heart failure” (5.73, 2015–2024), “toxicity” (3.16, 2018–2024), “neoadjuvant chemotherapy” (4.04, 2018–2024), and “apoptosis” (2.96, 2022–2024), all of which continue to show strong bursts through 2024.

## Discussion

This bibliometric cluster analysis identified four distinct research hotspots in epirubicin-induced cardiotoxicity: mechanisms of cardiotoxicity, treatment regimens, combination therapies, and drug formulations. Geographically, the United States demonstrated the highest citation impact, reflecting its substantial influence in advancing this research field, while Italy led in publication volume with significant contributions from prominent researchers. Among key institutions, the analysis revealed that specialized cancer centers and major universities have been instrumental in driving research progress, with *Journal of Clinical Oncology* (H-index = 34, TC = 2,786) emerging as the premier platform for disseminating critical findings in this field.

### Hotspots cluster and frontiers transition

The keyword co-occurrence analysis identified four research clusters in epirubicin-induced cardiotoxicity, each representing a distinct research focus.

#### Cluster 1 (Red): Mechanisms of Cardiotoxicity

This cluster explores the molecular and cellular mechanisms underlying epirubicin-induced cardiac toxicity, including oxidative stress, apoptosis, and cardiac dysfunction. Key terms include “cardiac toxicity”, “congestive heart failure”, “oxidative stress”, “doxorubicin-induced cardiotoxicity” and “trastuzumab”. Studies have highlighted iron-mediated ROS formation and mitochondrial damage as key contributors, with dexrazoxane identified as a potential cardioprotective agent (Sterba et al., 2013). Additionally, research on oxidative stress–mitochondrial dysfunction–ferroptosis genes has identified their role in immune infiltration and angiogenesis suppression, aiding in diagnostic and therapeutic advancements (Qianqian et al., 2024). Disruption of ATP6V0A2-dependent lysosomal acidification has also been linked to ferroptosis in cardiomyocytes, suggesting a potential intervention strategy (Zhang et al., 2024). The mechanistic insights from this cluster have directly influenced clinical practice through the development of biomarker-based early detection strategies and informed the design of novel cardioprotective agents beyond dexrazoxane. These findings have also contributed to updated clinical guidelines for cardiac monitoring and are driving the development of personalized treatment approaches based on individual genetic risk profiles.

#### Cluster 2 (Green): Chemotherapy Regimens and Clinical Management

This cluster examines epirubicin-based chemotherapy regimens, balancing efficacy with cardiotoxic risk. Keywords in this category include “cyclophosphamide”, “paclitaxel”, “randomized trial”, “efficacy” and “survival”. Six cycles of docetaxel and cyclophosphamide have been shown to be noninferior to EC-T in HER2-negative early breast cancer, supporting docetaxel and cyclophosphamide as a viable anthracycline-free option (Nitz et al., 2019). Immunomodulatory effects also differ, as epirubicin/cyclophosphamide (EC) suppresses lymphocytes, whereas docetaxel enhances immune responses, which may impact immunotherapy combinations (Wimmer et al., 2023). Additionally, epirubicin plus paclitaxel is noninferior to EC in disease-free survival for operable ERBB2-negative, ymph node-positive breast cancer, though with a higher toxicity profile (Yuan et al., 2023). While trastuzumab exacerbates anthracycline-related cardiac damage (An and Sheikh, 2019), meta-analyses indicate that its concurrent use with epirubicin enhances pCR rates in HER2-positive breast cancer without significantly increasing cardiotoxicity, though caution is needed in hormone receptor-positive cases (Yang et al., 2024). This research has directly transformed clinical practice by providing evidence-based alternatives to traditional anthracycline-containing regimens, which have been incorporated into major clinical guidelines (NCCN, ESMO). These findings are particularly valuable for elderly patients and those with pre-existing cardiac conditions, enabling the development of risk-adapted treatment algorithms that guide regimen selection based on individual patient characteristics.

#### Cluster 3 (Blue): Combination Therapies and Pharmacokinetics

This cluster focuses on the interaction of epirubicin with other chemotherapeutic agents and its pharmacokinetic properties, including “combination chemotherapy”, “colony-stimulating factor”, “cisplatin” and “phase II trial”. A phase II study demonstrated that weekly epirubicin and paclitaxel with G-CSF is well-tolerated and achieves a high overall response rate in metastatic breast cancer (Nistico et al., 2007). Similarly, a retrospective study found a higher-than-expected febrile neutropenia incidence (23.9%) in Japanese patients receiving EC therapy, advocating for prophylactic G-CSF use (Sakurada et al., 2019). A phase III trial confirmed that weekly cisplatin, epirubicin, and paclitaxel achieved significantly higher pCR rates than triweekly epirubicin and paclitaxel in locally advanced breast cancer, particularly in ER-negative and HER2-positive cases, though with increased toxicity (Frasci et al., 2006). Research in this cluster has led to improved supportive care protocols and population-specific dosing guidelines, particularly regarding ethnic differences in drug metabolism. The evidence supporting prophylactic G-CSF use has been incorporated into clinical practice guidelines, reducing treatment delays and hospitalizations. These findings have also influenced the design of adaptive dosing strategies in clinical trials.

#### Cluster 4 (Yellow): Drug Formulations and Cardiotoxicity Mitigation

This cluster explores alternative formulations and drug modifications aimed at reducing epirubicin’s cardiotoxicity, with key terms including “adriamycin”, “high-dose epirubicin”, “encapsulated doxorubicin” and “reduced cardiotoxicity”. Liposomal doxorubicin (Myocet^®^) has been shown to mitigate cardiotoxicity by reducing myocardial drug accumulation and upregulating DNA damage resistance pathways, making it a promising safer alternative (Gyongyosi et al., 2020). Additionally, surface-decorated PLGA nanoparticles significantly enhance oral bioavailability, achieving improved tumor suppression compared to free epirubicin (Tariq et al., 2016). A fucoidan-coated polymeric nanoparticle formulation encapsulating epirubicin has demonstrated superior anticancer efficacy in colorectal cancer, with reduced cardiotoxicity, supporting its potential as a safer chemotherapeutic option (Khan et al., 2024). This cluster represents successful translational research, with liposomal anthracyclines now approved and used as standard options for high-risk patients. The development of targeted delivery systems has influenced pharmaceutical industry investment in nanomedicine platforms and established new regulatory pathways for complex drug products. These advances are laying the foundation for next-generation smart drug delivery systems with enhanced cardioprotective capabilities.

### Evolution of study trends

An analysis of citation bursts reveals a clear shift in research priorities over time, transitioning from early study on chemotherapy efficacy to optimizing treatment strategies and, more recently, focusing on the mitigation and prevention of cardiotoxicity.

From 2004 to 2013, research primarily focused on chemotherapy protocols and their clinical effectiveness, particularly in breast cancer treatment. Keywords such as “combination” (2004–2009), “combination chemotherapy” (2004–2006), and “metastatic breast cancer” (2004–2009) highlight the growing emphasis on combination therapies to improve patient outcomes. During this period, anthracyclines, including epirubicin, were increasingly incorporated into standard regimens, with studies assessing their efficacy in both adjuvant and metastatic settings (Ardizzoia et al., 2007; Ejlertsen et al., 2004).

Since 2014, the research focused gradually shifted from treatment efficacy toward recognizing and addressing anthracycline-induced cardiotoxicity. The citation bursts of “anthracyclines” (2009–2015), and “heart failure” (2015–2024) indicate an increasing concern about the long-term cardiac effects of anthracycline-based therapies. Studies during this period examined mechanisms of cardiac dysfunction, oxidative stress, and myocardial injury, paving the way for improved risk assessment and early detection strategies (Mahmood et al., 2018). Articles focused on the long-term risk of heart failure in cancer patients treated with anthracycline-based chemotherapy, emphasizing predictive modeling and clinical trial insights (Fogarassy et al., 2019; Banke et al., 2018). The prominence of keywords such as “neoadjuvant chemotherapy” (2018–2024), “toxicity” (2018–2024), “heart failure” (2015–2024), and “apoptosis” (2022–2024) reflect efforts to develop safer chemotherapy regimens, investigate cardioprotective strategies, and understand cellular mechanisms underlying epirubicin-induced heart damage. Neoadjuvant chemotherapy has gained attention as a means of improving treatment outcomes while balancing toxicity risks (van der Voort et al., 2021; Zhang et al., 2022), while studies on apoptosis and oxidative stress have provided deeper insights into cardiac injury mechanisms, guiding potential therapeutic interventions (Al Khafaji et al., 2025; Wang et al., 2022).

### Strengths and limitations

This bibliometric analysis successfully identified four primary clusters, particularly in mechanisms of cardiotoxicity, chemotherapy regimens, combination therapies, and cardioprotective strategies. However, this study has several limitations. Database coverage bias may exist as this analysis was limited to WoSCC, potentially excluding relevant studies indexed in other databases such as Scopus or PubMed. The restriction to English-language publications may have excluded important research from non-English sources, particularly from countries with significant contributions to oncology research. The citation analysis was conducted with a cutoff date of December 31, 2024, which may not reflect the most recent citation patterns. Additionally, the exclusion of review articles, while methodologically sound, may have overlooked important synthesis studies that could influence research hotspot identification. Citation metrics may not fully capture clinical significance, and bibliometric methods focus on quantitative measures and may not assess the quality or real-world impact of studies. Future research should address these gaps by incorporating multilingual databases, extending the analysis to multiple database sources, and clinical outcome-based evaluations.

## Data Availability

The original contributions presented in the study are included in the article/Supplementary Material, further inquiries can be directed to the corresponding author.
