# Supplementary material for: Cluster analysis of hotspots and research trends of epirubicin-induced cardiotoxicity: a bibliometric study
Source: Front Pharmacol. 2025 Aug 20;16:1616162. doi: 10.3389/fphar.2025.1616162 (PMC12405342; doi:10.3389/fphar.2025.1616162)
Supplement: Supplementary file 1 [file Supplementaryfile1.docx]

**Table 1 Publication and Citation Profiles of Leading Countries**

| **Country** | **Articles** | **Freq** | **SCP** | **MCP** | **MCP_Ratio** | **TP** | **TP_rank** | **TC** | **TC_rank** | **Average Citations** |
| --- | --- | --- | --- | --- | --- | --- | --- | --- | --- | --- |
| ITALY | 129 | 19.2 | 117 | 12 | 0.093 | 590 | 2 | 3926 | 2 | 30.4 |
| CHINA | 93 | 13.8 | 85 | 8 | 0.086 | 368 | 3 | 1193 | 7 | 12.8 |
| USA | 53 | 7.9 | 44 | 9 | 0.170 | 226 | 5 | 12689 | 1 | 239.4 |
| FRANCE | 37 | 5.5 | 29 | 8 | 0.216 | 228 | 4 | 1651 | 5 | 44.6 |
| UK | 30 | 4.5 | 23 | 7 | 0.233 | 146 | 7 | 3047 | 3 | 101.6 |
| GERMANY | 27 | 4 | 22 | 5 | 0.185 | 191 | 6 | 1686 | 4 | 62.4 |
| JAPAN | 23 | 3.4 | 22 | 1 | 0.043 | 62 | 13 | 645 | 10 | 28 |
| NETHERLANDS | 20 | 3 | 14 | 6 | 0.300 | 128 | 8 | 742 | 8 | 37.1 |
| DENMARK | 17 | 2.5 | 12 | 5 | 0.294 | 85 | 10 | 1339 | 6 | 78.8 |
| CANADA | 15 | 2.2 | 12 | 3 | 0.200 | 77 | 11 | 645 | 9 | 43 |
| GREECE | 15 | 2.2 | 14 | 1 | 0.067 | 63 | 12 | 461 | 13 | 30.7 |
| BELGIUM | 13 | 1.9 | 4 | 9 | 0.692 | 97 | 9 | 355 | 14 | 27.3 |
| TURKEY | 13 | 1.9 | 13 | 0 | 0.000 | 42 | 17 | 140 | 21 | 10.8 |
| INDIA | 10 | 1.5 | 7 | 3 | 0.300 | 27 | 24 | 199 | 18 | 19.9 |
| SPAIN | 10 | 1.5 | 6 | 4 | 0.400 | 57 | 14 | 486 | 12 | 48.6 |
| AUSTRALIA | 9 | 1.3 | 7 | 2 | 0.222 | 44 | 15 | 233 | 17 | 25.9 |
| FINLAND | 9 | 1.3 | 8 | 1 | 0.111 | 37 | 21 | 166 | 19 | 18.4 |
| ROMANIA | 9 | 1.3 | 9 | 0 | 0.000 | 40 | 19 | 129 | 22 | 14.3 |
| NORWAY | 8 | 1.2 | 4 | 4 | 0.500 | 41 | 18 | 163 | 20 | 20.4 |
| SWITZERLAND | 7 | 1 | 2 | 5 | 0.714 | 37 | 22 | 571 | 11 | 81.6 |

Note(s): Articles: Publications of Corresponding Authors only. Freq: Frequence of Total Publications. SCP: Single Country Publications. MCP: Multiple Country Publications. MCP_Ratio: Proportion of Multiple Country Publications. TP: Total Publications. TP_rank: Rank of Total Publications. TC: Total Citations. TC_rank: Rank of Total Citations. Average Citations: The average number of citations per publication.

**Table 2 Bibliometric Indicators of High-Impact Journals**

| **Journal** | **h_index** | **g-index** | **m-index** | **TP** | **TP_rank** | **TC** | **TC_rank** | **PY_start** | **IF_2023** | **JCR_2023** |
| --- | --- | --- | --- | --- | --- | --- | --- | --- | --- | --- |
| JOURNAL OF CLINICAL ONCOLOGY | 34 | 39 | 0.829 | 39 | 1 | 2786 | 1 | 1985 | 42.1 | 1 |
| ANNALS OF ONCOLOGY | 24 | 35 | 0.800 | 35 | 2 | 699 | 2 | 1996 | 56.7 | 1 |
| EUROPEAN JOURNAL OF CANCER | 17 | 24 | 0.472 | 24 | 3 | 315 | 10 | 1990 | 7.6 | 1 |
| BREAST CANCER RESEARCH AND TREATMENT | 12 | 22 | 0.353 | 22 | 4 | 228 | 17 | 1992 | 3.0 | 2 |
| BRITISH JOURNAL OF CANCER | 12 | 18 | 0.333 | 18 | 6 | 296 | 11 | 1990 | 6.4 | 1 |
| CANCER CHEMOTHERAPY AND PHARMACOLOGY | 12 | 18 | 0.324 | 18 | 7 | 332 | 8 | 1989 | 2.7 | 2 |
| AMERICAN JOURNAL OF CLINICAL ONCOLOGY-CANCER CLINICAL TRIALS | 9 | 16 | 0.243 | 16 | 8 | 124 | 27 | 1989 | 1.6 | 4 |
| ANTICANCER RESEARCH | 9 | 13 | 0.257 | 20 | 5 | 110 | 31 | 1991 | 1.6 | 4 |
| ANTI-CANCER DRUGS | 8 | 15 | 0.229 | 15 | 9 | 85 | 37 | 1991 | 1.8 | 3 |
| JOURNAL OF PHARMACOLOGY AND EXPERIMENTAL THERAPEUTICS | 7 | 8 | 0.368 | 8 | 12 | 80 | 39 | 2007 | 3.1 | 2 |
| ONCOLOGY | 7 | 8 | 0.200 | 8 | 13 | 72 | 46 | 1991 | 2.5 | 3 |
| CANCER | 6 | 8 | 0.214 | 8 | 11 | 283 | 12 | 1998 | 6.1 | 1 |
| ONCOLOGIST | 6 | 7 | 0.240 | 7 | 14 | 130 | 25 | 2001 | 4.8 | 1 |
| BMC CANCER | 5 | 6 | 0.238 | 6 | 15 | 57 | 61 | 2005 | 3.4 | 2 |
| BREAST | 5 | 9 | 0.161 | 9 | 10 | 67 | 49 | 1995 | 5.7 | 1 |
| CLINICAL BREAST CANCER | 5 | 6 | 0.278 | 6 | 16 | 51 | 71 | 2008 | 2.9 | 2 |
| INTERNATIONAL JOURNAL OF CARDIOLOGY | 5 | 5 | 0.263 | 5 | 20 | 52 | 67 | 2007 | 3.2 | 2 |
| PLOS ONE | 5 | 6 | 0.385 | 6 | 17 | 83 | 38 | 2013 | 2.9 | 1 |
| ACTA ONCOLOGICA | 4 | 4 | 0.138 | 4 | 22 | 75 | 44 | 1997 | 2.7 | 3 |
| BIOMEDICINE & PHARMACOTHERAPY | 4 | 4 | 0.190 | 4 | 23 | 28 | 114 | 2005 | 6.9 | 1 |

Note(s): H_index: The H-index of the journal, which measures both the productivity and citation impact of the publications. IF: Impact Factor, indicating the average number of citations to recent articles published in the journal. JCR_Quartile: The quartile ranking of the journal in the Journal Citation Reports, indicating the journal's ranking relative to others in the same field (Q1: top 25%, Q2: 25%-50%, Q3: 50%-75%, Q4: bottom 25%). PY_start: Publication Year Start, indicating the year the journal started publication. TP: Total Publications. TP_rank: Rank of Total Publications. TC: Total Citations. TC_rank: Rank of Total Citations.

**Table 3** Publication and Citation Profiles of High-Impact Authors

| **Authors** | **h_index** | **g-index** | **m-index** | **PY_start** | **TP** | **TP_Frac** | **TP_rank** | **TC** | **TC_rank** |
| --- | --- | --- | --- | --- | --- | --- | --- | --- | --- |
| CONTE PF | 9 | 12 | 0.30 | 1996 | 12 | 1.35 | 1 | 518 | 10 |
| HOPEWELL JW | 8 | 8 | 0.21 | 1988 | 8 | 2.05 | 6 | 247 | 32 |
| SALVADORI B | 8 | 10 | 0.28 | 1997 | 10 | 1.06 | 3 | 359 | 15 |
| DANESI R | 7 | 8 | 0.19 | 1990 | 8 | 0.79 | 5 | 338 | 21 |
| GENNARI A | 7 | 10 | 0.24 | 1997 | 10 | 1.45 | 2 | 511 | 12 |
| NAMER M | 7 | 7 | 0.28 | 2001 | 7 | 0.63 | 14 | 558 | 8 |
| ROSSO R | 7 | 8 | 0.20 | 1991 | 8 | 0.76 | 8 | 1771 | 1 |
| ABNOUS KHALIL | 6 | 6 | 0.46 | 2013 | 6 | 0.79 | 18 | 336 | 22 |
| BALDINI E | 6 | 7 | 0.21 | 1997 | 7 | 0.62 | 9 | 259 | 30 |
| BENGALA C | 6 | 9 | 0.21 | 1998 | 9 | 0.97 | 4 | 202 | 38 |
| BRUZZI P | 6 | 6 | 0.20 | 1996 | 6 | 0.61 | 19 | 539 | 9 |
| CADEDDU CHRISTIAN | 6 | 6 | 0.32 | 2007 | 6 | 0.71 | 20 | 355 | 16 |
| DEL TACCA M | 6 | 6 | 0.22 | 1999 | 6 | 0.61 | 22 | 235 | 35 |
| DESSI MARIELE | 6 | 6 | 0.32 | 2007 | 6 | 0.71 | 23 | 355 | 16 |
| DONATI S | 6 | 7 | 0.22 | 1999 | 7 | 0.70 | 10 | 157 | 45 |
| FARGEOT P | 6 | 6 | 0.18 | 1993 | 6 | 0.41 | 24 | 584 | 5 |
| FUMOLEAU P | 6 | 6 | 0.18 | 1993 | 6 | 0.41 | 25 | 584 | 5 |
| GUARNERI V | 6 | 7 | 0.24 | 2001 | 7 | 0.64 | 11 | 136 | 47 |
| KERBRAT P | 6 | 6 | 0.18 | 1993 | 6 | 0.41 | 26 | 584 | 5 |
| LOPEZ M | 6 | 8 | 0.17 | 1991 | 8 | 1.00 | 7 | 250 | 31 |

Note(s): H_index: The H-index of the author, measuring both productivity and citation impact of their publications. G_index: The G-index of the author, giving more weight to highly-cited articles. M_index: The m-index of the author, calculated as the h-index divided by the number of years since their first published paper. PY_start (Publication Year Start): The year in which the author’s first publication appeared. TP (Total Publications): The total number of publications by the author. TP_rank (Rank of Total Publications): The author's rank based on the total number of publications. TC (Total Citations): The total number of citations received by the author's publications. TC_rank (Rank of Total Citations): The author's rank based on the total number of citations. Average Citations: The average number of citations per publication for the author.
